# Comparison of Enhanced Noise Model Performance Based on Analysis of Civilian GPS Data

**DOI:** 10.3390/s20216050

**Published:** 2020-10-24

**Authors:** Andy W. R. Soundy, Bradley J. Panckhurst, Phillip Brown, Andrew Martin, Timothy C. A. Molteno, Daniel Schumayer

**Affiliations:** Department of Physics, University of Otago, 730 Cumberland St, Dunedin 9016, New Zealand; souan326@student.otago.ac.nz (A.W.R.S.); panbr907@student.otago.ac.nz (B.J.P.); phillb@elec.ac.nz (P.B.); andrew.martin@otago.ac.nz (A.M.); tim@physics.otago.ac.nz (T.C.A.M.)

**Keywords:** noise models, stochastic process, GPS error, Ornstein–Uhlenbeck process, sensor fusion, embedded computing

## Abstract

We recorded the time series of location data from stationary, single-frequency (L1) GPS positioning systems at a variety of geographic locations. The empirical autocorrelation function of these data shows significant temporal correlations. The Gaussian white noise model, widely used in sensor-fusion algorithms, does not account for the observed autocorrelations and has an artificially large variance. Noise-model analysis—using Akaike’s Information Criterion—favours alternative models, such as an Ornstein–Uhlenbeck or an autoregressive process. We suggest that incorporating a suitable enhanced noise model into applications (e.g., Kalman Filters) that rely on GPS position estimates will improve performance. This provides an alternative to explicitly modelling possible sources of correlation (e.g., multipath, shadowing, or other second-order physical phenomena).

**Dataset License:** BY-NC-ND

## 1. Introduction

Numerous applications are built using location provided by Global Positioning System (GPS) receivers. While centimetre precision has been demonstrated for dual-frequency [1] and differential GPS systems, a very large number of systems rely on single-frequency non-differential units, which report positions with typical precision of many metres [2]. We use the term “noise” to refer to the errors in a sequence of reported positions. The sources of the noise in the positions include atmospheric and ionospheric phenomena [3], multipath effects [4,5], canopy trees [6], and a wide variety of physical phenomena that may be impractical to model. However, other types of errors are also inadvertently added to the signal, e.g., errors present in the modelling of satellite orbits [7], finite precision of the digital components of receivers, variations in the built-in phased-locked loop [8], or the signal processing techniques [9], etc. The common structure and frequent formulations of GPS algorithms are discussed in Xu and Xu [10], including some aspects of the mathematical formulation that carry non-trivial correlation in readings between epochs. In summary, however, it is sufficient for this work to realise that there are noise sources that can be adequately modelled by uncorrelated processes, e.g., white noise, and there are sources that inherently carry temporal correlations.

All GPS signals are encumbered with noise, whose sources are hard to distinguish [11,12]. The noise could be attributed to satellite clock and orbit errors, to the propagation of the GPS signal through media (ionosphere, troposphere, etc.), and to the receiver units. Many of these noise components are time-correlated and caused by stochastic processes external to the useful signal. It would be ambitious to model and distinguish between all these processes; alternatively, one can consider their cumulative effect on the time series [13,14]. Irrespective of the source, a robust signal-processing algorithm needs some model of noise processes. Such a model unavoidably contains assumptions on the nature of the noise, e.g., amplitude and correlation in the time domain, which must be experimentally validated. Often, the measurement errors are modelled by independent, identically distributed random Gaussian variables (Gaussian white noise). While this model is sufficient for many applications, it is generally accepted that a linear combination of coloured noise (whose power spectrum follows a $P∼f^{-\kappa }$ law with a characteristic exponent κ) is a more suitable approximation [15]. With the assumption of white noise, the time correlation in GPS datastreams leads to biased and inconsistent parameter estimates [16], especially for sampling rates above 5 Hz. Moreover, it is unrealistic that measurements from different satellites would have the same accuracy; therefore, stochastic models assuming independent and constant variance result in unreliable statistics, and stochastic modelling is needed. Directly estimating statistical parameters of the GPS time series by explicitly taking into account the heteroscedastic space- and time-correlated error structure of the observations could result in improved accuracy at minimal computational cost.

Noise in long GPS time series has been analysed for geodetic purposes by [17,18], who found −1≤κ≤3 in the relevant frequency band. Their results are, however, not conclusive, as many spectra would fit this range, e.g., white noise (κ=0) or flicker noise (κ=1). Many processes that these GPS observations are expected to capture, e.g., earthquakes or the wobble of the Earth around its axis, have similar spectra [19]. Consequently, the physical process being observed by GPS and the inherent noise of a GPS signal are present simultaneously. The noise amplitude changes with time and, for older data, can be a considerable fraction of the signal [20].

In this study, we analyse time series of GPS measurements with duration between a day and a week at 1 Hz sampling frequency. Observations were made in the Southern Hemisphere with stationary units. The recordings show significant autocorrelation incompatible with a Gaussian white noise model. The observed presence of correlations implies that there are some stochastic equations governing the dynamics of the signal. There are two major sources that plausibly introduce correlations: (a) the movement of GPS satellites and, thus, their constellation, and (b) the algorithms coded in the GPS emitters and receivers. Both sources are, to a high degree, deterministic; thus, in a perfect environment, they would produce a strongly correlated signal. However, there are many other phenomena, e.g., weather and surface reflectance, that introduce effects into the signal that can be most effectively modelled by noise, rather than searching for their respective governing equations. We develop mathematical models for the observed noise and suggest how these models can be used for improved positioning and uncertainty quantification in future devices.

## 2. Description of the Data Collection Process

Data were collected from civilian L1-band GPS units at multiple locations. At each location, data were collect from four units with a Telit Jupiter-F2 integrated GPS module, as well as a single standard USB-connected GPS receiver—GlobalSat BU-353S4. The first- and second-order statistics of the location data showed no significant difference between the two devices. For brevity, we present our findings using the data obtained only with the GlobalSat BU-353S4.

The location of satellites is a major factor of GPS accuracy degradation. Devices function well when the satellites are widely distributed [21]; hence, the altitude errors in measurements are typically much larger than those in latitude or longitude. The GPS satellites operate at an inclination of around 55${}^{\circ }$; consequently, for devices at higher latitudes, the GPS satellite positions are biased strongly towards the equator.

**Figure 1:**
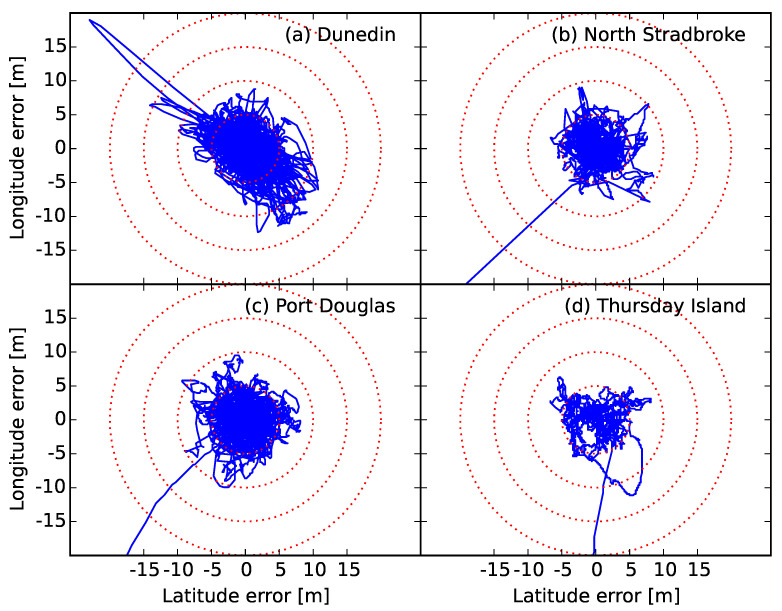
The longitude and latitude errors at each location. Due to the large number of observations, the data have been decimated.

To ensure that our analysis was not biased due to specifics of the GPS satellite constellation geometry, we carried out measurements at four locations with latitudes between 45${}^{\circ }$ and 10${}^{\circ }$ South (see Table 1). Data were recorded at each site for approximately 48 h. The decimated latitude and longitude errors (deviations from time average) are shown in Figure 1. Disregarding the initialisation period (typically ∼1 min), the data resemble a random walk.

While this cursory look seems suggestive, calculating the residuals defined as $r_{t}=X_{t}-\xi_{0}$ (here, $\xi_{0}$ denotes the long-time average, or the ‘true’ position) and creating a QQ-plot [22] reveals the non-Gaussian nature of $r_{t}$ (for technical details see Appendix A).

**Figure 2:**
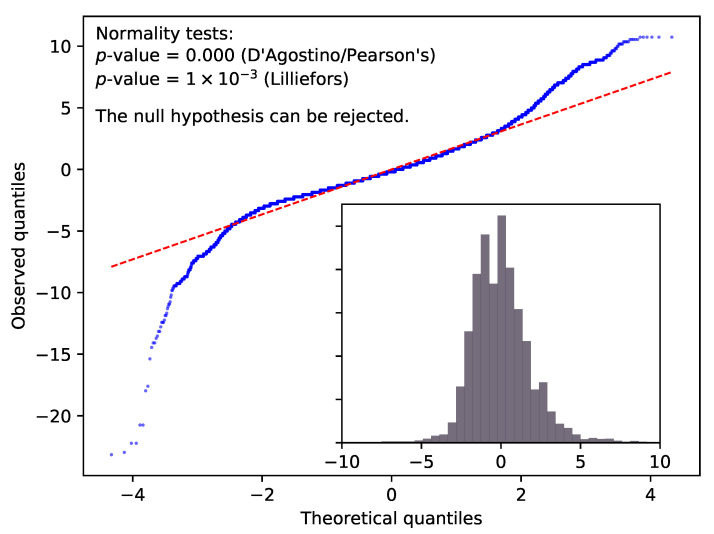
The main figure shows the QQ-plot of the residual of latitude data (measured in metres) collected in Dunedin. The inset depicts the histogram of the same data. The *p*-values of two normality tests are also given in the top left corner. Based on this evidence, the data do not support the assumption that the residual follows a Gaussian distribution.

In Figure 2 the QQ-plot of the latitude data collected in Dunedin is depicted. In order to support the graphical findings, we have also calculated two more normality tests [23], one based on d’Agostino and Pearson’s work [24,25], and another one suggested by Lilliefors [26]. The latter is the most suitable for our use, as it does not assume the knowledge of mean and standard deviation of the normal distribution, but estimates these from the sample. The null hypothesis of both tests is that the data come from a normal distribution. Since both tests return *p*-values much smaller than 0.01, we can thus conclude at a 99% confidence level that latitude data do not support the assumption of normal distribution. Similar conclusions can be drawn for longitude and altitude data at all locations.

## 3. Analysis of Observations

One may think of the collection of positions—emitted by the GPS chip—as a stochastic process, a sequence of random variables, $X_{1},X_{2},\;\cdots ,X_{t},\cdots$ labelled by time t=1,2,⋯ In our study, $X_{t}$ may represent the latitude, longitude, or altitude returned by a GPS unit. We denote random variables with capital letters, e.g., $X_{t}$, and its particular realisation with lower case letter, e.g., $x_{t}$. Any estimator of a statistic, e.g., the expectation value μ, is denoted by the same letter but decorated with a m^ symbol, e.g., $\hat{\mu }$.

### Stochastic Processes

The simplest non-trivial stochastic process can be formulated as the sum of the true position, $\xi_{0}$, and an additive noise term:

(1) $$X_{t}=\xi_{0}+E_{t}.$$

The measurement error, $E_{t}$, is often considered to be a Gaussian white noise with zero mean and variance $\sigma^{2}$. The problems of fitting Gaussian noise models to GPS measurements have been recognised by [27,28], and two models were proposed to describe the measurements more accurately: a moving average process (MA) and an autoregressive model (AR).

We check whether adjacent recordings can be considered independent random variables using the empirical autocorrelation function [29,30,31,32,33], defined as

(2) $$\hat{\rho }\left(t,s\right)=\frac{\mathrm{cov}\;\left(X_{t},X_{s}\right)}{\sigma_{t}\sigma_{s}}=\frac{E\left[\left(X_{t}-\mu_{t}\right)\left(X_{s}-\mu_{s}\right)\right]}{\sigma_{t}\sigma_{s}},$$

where $\mu_{r}$ and $\sigma_{r}$ are the expectation value and variance of the random process at step *r*, respectively. The process $\left\{X_{t}\right\}$ is stationary if $\mu_{t}$ and $\sigma_{t}$ are both time-independent; in such a case, the autocorrelation function depends only on the time difference h=s−t (*lag*) between its arguments and not explicitly on the arguments themselves. Henceforth, we assume stationarity; thus, ρ(t,s)=ρ(h) while $\mu_{t}=\mu$ and $\sigma_{t}=\sigma$ both become constants, which are calculated by time-averaging. Furthermore, we consider the empirical partial autocorrelation function calculated using the Yule–Walker method [32,34] and defined as

(3) $${\hat{\rho }}_{p}\left(h\right)=\frac{\mathrm{cov}\;\left(X_{t},X_{t+h}|X_{t+1},X_{t+2},\cdots ,X_{t+h-1}\right)}{\sigma_{t}\sigma_{t+h}},$$

which provides the amount of correlation between a variable and a lag of itself that is not explained by correlations at all lower-order lags.

**Figure 3:**
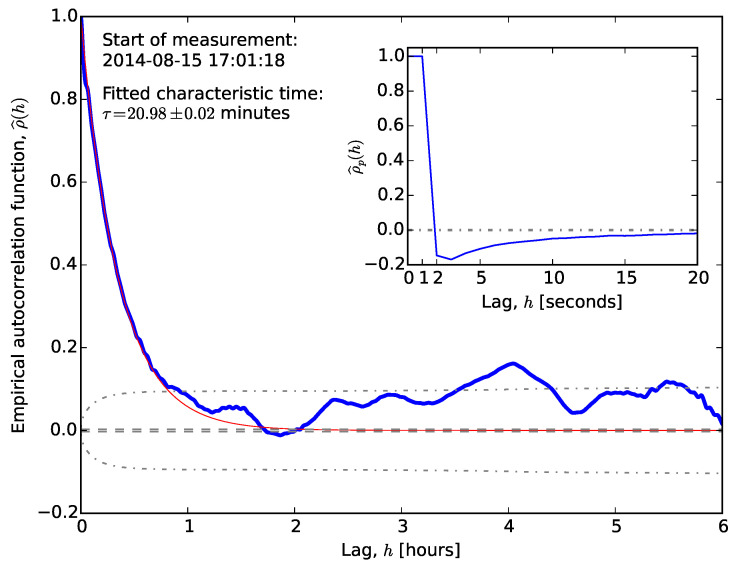
Correlogram of latitude errors of a time series from Dunedin of ∼250 h. The dashed lines correspond to the classical 95% confidence interval, while the dash-dotted curve corresponds to the Box–Jenkins standard error. The thin solid line represents the exponentially decaying curve fitted to the first half-hour section. The inset depicts the empirical partial autocorrelation function.

As an example, Figure 3 shows the autocorrelation of a typical time series. The autocorrelation function decays slowly. Fitting an exponential decay to the first half-hour dataset provides a characteristic time estimate of τ=20.98±0.02 minutes, i.e., it takes approximately an hour (∼3τ) to reach statistical independence. Using the reported position for periods much shorter than τ would lead to a biased estimate. We wish to draw the reader’s attention to the fact that long-term correlations usually imply a power-law decay of correlation, while our fit assumes an exponential correlation. While exponential decay of correlation is relatively fast, compared to power-law decay, it seems to have an observable effect on location time series, and its inclusion in modelling may provide better positioning algorithms in the future.

The theoretical autocorrelation function of Gaussian white noise is the Dirac-delta, ρ(t,s)∝δ(t−s). Assuming this model, one may test for statistical independence of $X_{t}$ and $X_{t+h}$ by checking whether the autocorrelation function is different from zero, i.e., whether it falls within the confidence interval around $\hat{\mu }=0$ [35,36].

$$-\frac{z\;\left(1-\frac{\alpha }{2}\right)}{\sqrt{n}}\;\hat{\sigma }\leq \hat{\rho }\left(h\right)\leq \frac{z\;\left(1-\frac{\alpha }{2}\right)}{\sqrt{n}}\;\hat{\sigma },$$

where z(x) is the cumulative distribution function of the standard normal probability distribution, α is the required level of significance, $\hat{\sigma }$ denotes the sample standard deviation, and *n* is the number of measurements. This confidence interval at the α=0.05 level is plotted with dashed lines in Figure 3. The majority of $\hat{\rho }\left(h\right)$ falls outside this interval; therefore, our observations and the assumption of Gaussian white noise are inconsistent with each other. One may take into account that the empirical autocorrelation function estimated from a finite-length time series also carries error [37] and could derive a modified confidence interval. At significance level α, this is given by the Box–Jenkins standard error [38,39,40]:

$$-\frac{z\;\left(1-\frac{\alpha }{2}\right)}{\sqrt{n}}\;S\left(h\right)\leq \hat{\rho }\left(h\right)\leq \frac{z\;\left(1-\frac{\alpha }{2}\right)}{\sqrt{n}}\;S\left(h\right),$$

where $S\left(h\right)=\sqrt{1+2\sum_{k=1}^{h-1}{\hat{\rho }}^{\;2}\left(k\right)}$. Figure 3 also depicts the Box–Jenkins standard error with dash-dotted lines, and it is apparent that the two confidence intervals differ from each other substantially, thus confirming the statistical dependence in the measured location readings. It is worth noting here that the exponential fit and the first crossing of $\hat{\rho }\left(h\right)$ with the Box–Jenkins confidence interval agree with each other. The inset in Figure 3 shows ${\hat{\rho }}_{p}\left(h\right)$ for h≤20 s with its standard error at a 0.95 significance level. This graph may suggest the application of a high-order autoregressive noise model (see next section), since $\hat{\rho_{p}}\left(h\right)$ vanishes only for lags larger than approximately 30 s.

However, we find that other datasets exhibit much shorter ‘memory’, so lower-order processes might also be sufficient.

**Figure 4:**
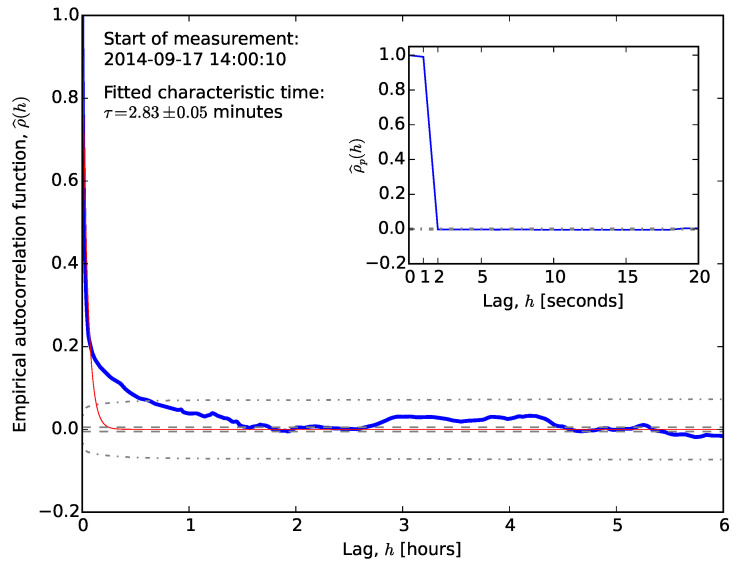
Correlogram of longitude errors for the GPS data taken in Port Douglas. The line styles are identical to those in Figure 3.

Datasets from other locations exhibited different behaviour (see Figure 4). The autocorrelation still takes less than an hour to reach the Box–Jenkins standard error, but the initial drop is much sharper. This drop may explain the different behaviour of the partial autocorrelation function, which vanishes for lags larger than unity. Figure 3 and Figure 4 illustrate the extreme cases.

**Figure 5:**
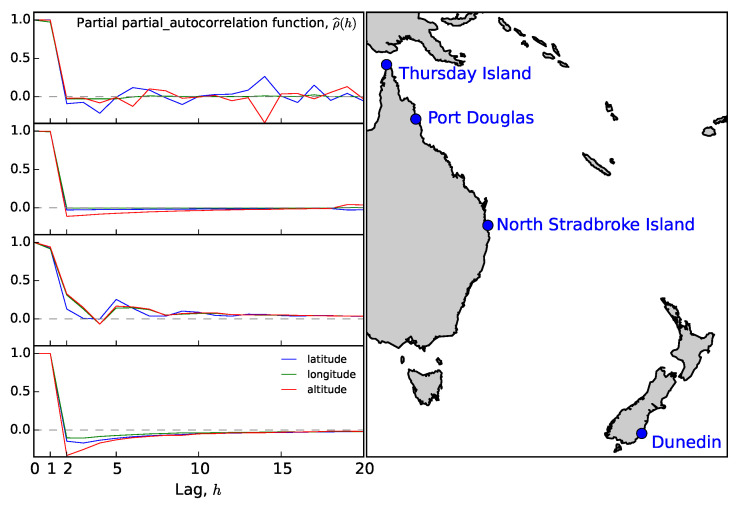
The partial autocorrelation function, ${\hat{\rho }}_{p}\left(h\right)$, for longitude, latitude, and altitude at all geographic locations visited. The lag is measured in seconds.

The autocorrelation of the Thursday Island dataset, see Figure 5, displays persistent oscillations not observed at other locations. This could be because of a temporary loss of power experienced on Thursday Island, which forced the data to be split into two sets, each approximately 24 h long, or an artefact of the local geography contributing to long-term multipath effects.

## 4. Modelling the Noise

In this section, we develop some statistical models for the observed noise. A model is a relationship between random variables, ${\left\{X_{t+k}\right\}}_{k\in K}$, in the following form:

(4) $$h\;\left({\left\{X_{t+k}\right\}}_{k\in K}\right)=g\;\left({\left\{E_{t+\ell }\right\}}_{\ell \in L}\right),$$

where K and L are sets of integers, *h* and *g* are fixed functions, and $E_{t}∼N\left(0,\sigma^{2}\right)$. Here, the quartet (*h*, *g*, K, L) constitutes the model to be inferred. To simplify the task, we presume that *h* and *g* are linear in all their variables (so-called linear models). The stochastic model is then

(5) $$\sum_{k=0}^{\infty }h_{k}X_{t-k}=\sum_{\ell =0}^{\infty }g_{\ell }E_{t-\ell },$$

where $h_{k}$ and $g_{\ell }$ are all constants with $h_{0}=g_{0}=1$ (convenience), and causality restricts summations to the current or past values of $X_{t}$ or $E_{t}$ [41]. This model requires an infinite number of coefficients, which cannot be estimated using a finite number of observations. Therefore, we focus on finite-parameter linear models and estimate the unknowns via the maximum likelihood method, in which the likelihood function below is constructed for Gaussian processes.

The likelihood function, *L*, is the joint probability density of obtaining the actually observed data $x=(x_{1}$, $x_{2}$, …, $x_{N})$, according to the model. It is a function of the unknown parameters $\beta =(h_{0}$, $h_{1}$, …, $g_{0}$, $g_{1}$, …), and $\sigma^{2}$. Under general assumptions, the log-likelihood function of a finite parameter linear model is:

$$\log\;\left(L(\beta ,\sigma^{2})\right)=-\frac{1}{2}\;\left[N\log\;(2\pi )\;+\;\log\;\left(\det\;\left(\sigma^{2}\;\Sigma \left(\beta \right)\right)\right)\;+\;\frac{1}{\sigma^{2}}x^{T}\Sigma^{-1}\left(\beta \right)\;x\right]$$

where the (m,n)-th component of the covariance matrix $\sigma^{2}\Sigma \left(\beta \right)$ is calculated from

$$\sigma^{2}\Sigma_{m,n}=\frac{\sigma^{2}}{2\pi }\int_{-\pi }^{\pi }\;\;e^{i(m-n)\omega }{\left|\frac{g_{0}+g_{1}z+g_{2}z^{2}+\cdots }{h_{0}+h_{1}z+h_{2}z^{2}+\cdots }\right|}_{z=e^{-i\omega }}^{2}\;d\omega .$$

Although the likelihood function, *L*, is derived under Gaussian processes, if the time series is causal and invertible, then maximising this likelihood leads to asymptotically consistent estimators [42,43]. This approximation is convenient, as the asymptotic likelihood function has a unique stationary point, the global maximum [44].

Below, we introduce and analyse finite-parameter linear noise models and compare their performance, including the Gaussian white noise model for a benchmark.

### 4.1. Gaussian White Noise Model

The simplest noise model treats measurements as statistically independent, normally distributed random variables: $X_{t}-\mu =E_{t}$ for fixed μ. Thus, $X_{t}∼N\left(\mu ,\sigma^{2}\right)$, with yet unknown parameter values μ and σ. Using the maximum likelihood approach, these parameters are estimated from the observations as

$$\hat{\mu }=\frac{1}{N}\sum_{i=1}^{N}x_{i}\;and\;{\hat{\sigma }}^{2}=\frac{1}{N}\sum_{i=1}^{N}{\left(x_{i}-\hat{\mu }\right)}^{2}.$$

### 4.2. Autoregressive Models

An autoregressive process of order *p*, abbreviated as AR(*p*), is a stochastic process where *h* is a linear combination of *p* terms:

$$X_{t}=-\sum_{k=1}^{p}h_{k}X_{t-k}+E_{t},$$

where $h_{k}$ are constants. The process has p+1 parameters: ${\left\{h_{k}\right\}}_{k=1}^{p}$ and σ. The estimators ${\hat{h}}_{i}$ and ${\hat{\sigma }}^{2}=\hat{\rho }\left(0\right)+\sum_{k=1}^{p}\hat{\rho }\left(k\right){\hat{h}}_{k}$ are found by linear algebra [37]. Alternatively, the estimators can be found by least squares regression [45]. The parameter estimation procedure costs, at most, $O({\left(p+1\right)}^{2}+np)$ operations, where *n* is the number of observations.

As the Laplace transformation of $X_{t}$ suggests the autocorrelation function of an AR(*p*) process is formally the sum of *p* exponentially decaying terms, thus, $\hat{\rho }\left(h\right)$ exhibits exponential decay or oscillatory behaviour [32], while ${\hat{\rho }}_{p}\left(h\right)$ vanishes for lags p<h.

### 4.3. Moving Average Model

Rather than using the past values of the random variable, a moving average model of order *q*, abbreviated as MA(*q*), regresses on past errors, $E_{t-k}=X_{t-k}-E\left(X_{t}\right)$ for k=0,⋯q, and is defined as

$$X_{t}=\mu +\sum_{\ell =0}^{q}g_{\ell }E_{t-\ell }.$$

The process has q+2 parameters. One may cast any stationary AR(*p*) process as an MA(*∞*) model and an invertible MA(*q*) model as an AR(*∞*) process. Nevertheless, MA(*q*) models are useful, as one cannot fit an AR(*∞*) model on a finite number of observations.

Unlike autoregressive processes, for MA processes, any estimator based on a finite amount of observations inherently carries some loss of information. Resultantly, parameter estimation is harder than for AR processes of comparable order.

The qualitative behaviours of the autocorrelation and partial autocorrelation functions, $\hat{\rho }\left(h\right)$ and $\hat{\rho_{p}}\left(h\right)$, are similar to those in autoregressive processes, but with roles interchanged. The autocorrelation function vanishes for h>q, while the partial autocorrelation function is dominated by damped exponential and/or periodic functions. In the examples shown in Figure 3 and Figure 4, the autocorrelation function vanishes too slowly to be described by a low-order MA process.

### 4.4. Autoregressive Moving Average Model

The combination of AR(*p*) and MA(*q*) processes can be formulated as

$$X_{t}=\mu -\sum_{k=1}^{p}h_{k}X_{t-k}+\sum_{\ell =0}^{q}g_{\ell }E_{t-\ell }.$$

The combination inherits most of its properties from these simpler processes. Neither the autocorrelation nor the partial autocorrelation function vanishes after a threshold lag. The autocorrelation function exhibits damped exponential and/or oscillatory behaviour for lags h>q−p, while $\hat{\rho_{p}}\left(h\right)$ is dominated by exponential and periodic terms for lag values h>p−q. The variance of the sample autocorrelation function can exceed the Box–Jenkins error (see Figure 3). As a result, it is often difficult to determine the model orders; model verification and iterative parameter estimation are needed.

For normally and independently distributed $E_{t}$ with zero mean and constant variance, and for stationary $\left\{X_{t}\right\}$, Melard [46] provided an efficient algorithm for calculating the exact likelihood function of an ARMA(*p*, *q*) model. This algorithm requires $O(p^{2}+q^{2}+n\left(p+3q\right))$ operations for *n* observations, which—in practice—can be reduced by considering the observations in a finite window.

### 4.5. Ornstein–Uhlenbeck Model

In this section, we consider a stochastic differential equation (SDE) rather than a discrete stochastic process. Stochastic partial differential equations are a natural choice to model dynamical systems subject to random fluctuation. For an introduction to SDEs, we point to Evans’ [47] and Øksendal’s [48] excellent monographs.

The scatter-plots in Figure 1 resemble a random walk on a phenomenological level. However, in a Brownian motion, the variance of the position increases with time, which we do not observe here. On the contrary, we observe (and expect) that the GPS reading will return to the true location even if it deviates from it momentarily, i.e., it is mean-reverting. Furthermore, Figure 3 shows that the correlation function decreases exponentially. These properties suggest an Ornstein–Uhlenbeck (OU) process [49], defined by the SDE

(6) $$dX_{t}=\theta \left(\mu -X_{t}\right)dt+\sigma dW_{t},$$

where θ, μ, and σ are real parameters, with θ,σ>0. For historical and physical reasons, the parameters θ and σ are called the drift and diffusion coefficients, respectively. The fluctuation part, $W_{t}∼N\left(0,t\right)$, is a Wiener process. The same process can also be represented as

$$X_{t}=X_{0}e^{-\theta t}+\mu \left(1-e^{-\theta t}\right)+\sigma e^{-\theta t}W_{(e^{-2\theta t}-1)/2\theta }.$$

The first term corresponds to the long-term but diminishing influence of the initial observation. The second reaches the steady value μ exponentially fast, while the third describes a scaled and time-transformed random fluctuation. The expectation value at time *t* is $E\left(X_{t}\right)=\mu \left(1-e^{-\theta t}\right)+e^{-\theta t}E\left(X_{0}\right)\approx \mu$.

One may simplify (6) by eliminating μ with the $X_{t}\mapsto X_{t}-\mu$ transformation, but we keep μ explicit, as it is the ‘true’ position of the GPS device. The parameter θ sets the scale of the autocorrelation function ρ(h), while σ determines the noise between measurements. The OU process is the continuous-time generalisation of an AR(1) process. It has several desirable properties: (a) It is mean-reverting for θ>0, (b) it has finite variance for all t≥0 contrary to Brownian motion, (c) its analytic covariance $\mathrm{cov}\;\left(X_{t},X_{u}\right)∼e^{-\theta \left|t-u\right|}$ permits a long autocorrelation time, (d) the autocorrelation function is insensitive to the sampling rate, and (e) it approaches Gaussian white noise as θ→∞. More precisely, the OU process is the only non-trivial, mean-reverting, Gauss–Markov process, i.e., whose transitions are memory-less, and it thus allows predictions of the future state based on the current state [50]. This does not prohibit more sophisticated models for GPS time series, but these models will lack at least one of the properties listed above.

To model our observations as an Ornstein–Uhlenbeck process, we must estimate θ, μ, and σ using maximum likelihood estimation [51]. Tang and Chen [52] provided a detailed derivation of estimators for these parameters, and Dacunha-Castelle and Florens-Zmirou [53] showed that for time-equidistant observations, the maximum likelihood estimators are consistent and asymptotically normal, irrespective of the sampling time-step, Δt.

The estimators for θ, μ, and σ require ∼2n operations each. Therefore, the overall parameter estimation takes O(6n) operations. However, Tang also gives explicit estimates for the asymptotic form of these estimators, e.g.,

$$E\;\left(e^{-\hat{\theta }\Delta t}\right)=e^{-\theta \Delta t}-\frac{4-3\theta \Delta t}{n}+O\;\left(\frac{1}{n^{2}}\right).$$

Thus, one needs only a moderate number of observations, *n*, or short sampling time Δt to estimate the θ parameter, especially compared with the estimator of the mean for an uncorrelated process, which would have $O(1/\sqrt{n})$ (much slower) convergence to the true mean.

Parameter estimation for SDEs is a rapidly developing field, especially the sub-field of online estimators that update the parameter estimates as newer observations become available. Such estimators are usually associated with constant computational cost, as their performance is independent of *n*. A method of online estimators for a sampled, continuous-time Ornstein–Uhlenbeck is the subject of future publication.

**Figure 6:**
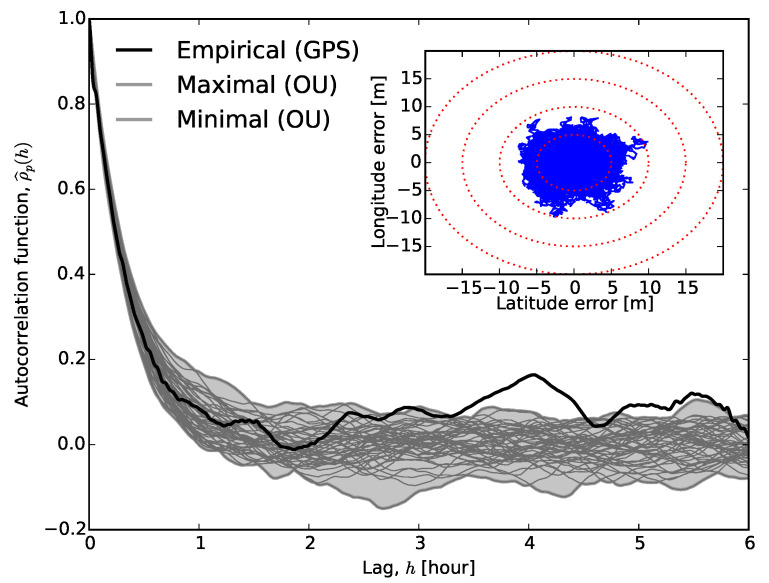
The thick black solid line depicts $\hat{\rho }\left(h\right)$ for the GPS recordings on 15 August, 2014 in Dunedin, as in Figure 3. All grey lines represent the autocorrelation function of a simulated Ornstein–Uhlenbeck process with input parameters $\mu =\hat{\mu }≅45.864$, $\theta =\hat{\theta }≅1.329\times 10^{-4}$, and $\sigma =\hat{\sigma }≅2.745\times 10^{-7}$. The lines labelled as ‘maximal’ and ‘minimal’ are connecting the extremal values of simulated autocorrelation functions at all lags.

After estimating μ, θ, and σ, we simulated an ensemble of Ornstein–Uhlenbeck processes using the Euler–Maruyama method [54]. We calculated and plotted the autocorrelation function for each run (see Figure 6) as well as the range covered by these functions. Clearly, the OU noise model represents the autocorrelation in the data up to at least two hours. However, it is unable to capture the undulation of $\hat{\rho }\left(h\right)$. Therefore, an Ornstein–Uhlenbeck process should be complemented with some other process for longer-term predictability. We believe that a more complicated noise model is unwarranted unless the sampling period exceeds an hour.

## 5. Quantitative Analysis of Noise Models

In this section, we present and discuss the quantitative performance of the previously described noise models. For each model, the parameters were chosen to best fit the location data. The process of selecting which of these models best describes the data, called model selection, is a large and complex field in statistics; thus, we leave detailed explanation to the technical literature, e.g., [55] and references therein.

There are three properties of model selection criteria: consistency, efficiency, and parsimony. Consistency means that the criterion selects the true model with probability approaching unity with increasing sample size provided the true model is among those considered. However, nothing guarantees that the true model is simple and tractable; thus, it is also necessary to measure how much error one might make by accepting a model knowing that it is not the true model. Efficiency is a measure of this property; as the sample size becomes large, the error in predictions using the selected model becomes indistinguishable from the error obtained using the best model among the candidates. Parsimony dictates that if all measures are equal for two competing statistical models, the preferred one should be the simpler model.

The most common model selection criteria use the likelihood function, *L*, and introduce some penalty proportional to the model complexity. Akaike’s Information Criterion (AIC) is defined as $\mathrm{AIC}=-2\ln\;\left(L\right)+2k$, where *k* is the number of fit parameters in the model [56,57]. The best model has the smallest AIC value—it encodes the most information using the least number of fitted parameters. The AIC has a definite minimum value with respect to *k*, and the appropriate order of the model is determined by the value of *k* at this minimum. The AIC is biased for small samples, and a bias-corrected version (AICc) is gaining popularity [43]. Here, we analyse time series with $n>10^{5}$ and k≈10, so we can ignore this correction. Other criteria have been developed, e.g., Bayesian Information Criterion (BIC), which more severely penalises the introduction of extra model parameters by replacing the 2k term with k+kln(n).

Regarding the model selection criteria, AIC and AICc are both inconsistent, while BIC is only asymptotically consistent. Meanwhile, AIC is efficient, while BIC is not. Therefore there is no clear choice between AIC and BIC. The latter is asymptotically consistent as the sample size approaches infinity, while the former selects models that have acceptable predictive power [58].

We use AIC, as it is almost universally accepted. While it does not validate a statistical model, it determines relative probabilities between alternative models. We compare AIC values for a selection of models. Table 2 shows AIC scores for model fits of latitude, longitude, and altitude at four geographic locations. For each location, we individually fitted different noise models using the same observation datasets. For the AR(*p*), MA(*q*), and the ARMA(*p*, *q*), we increased the number of parameters involved. Occasionally the statistics routine was unable to converge for the MA(*q*) processes. In these cases, the corresponding entry in Table 2 is left empty.

**Figure 7:**
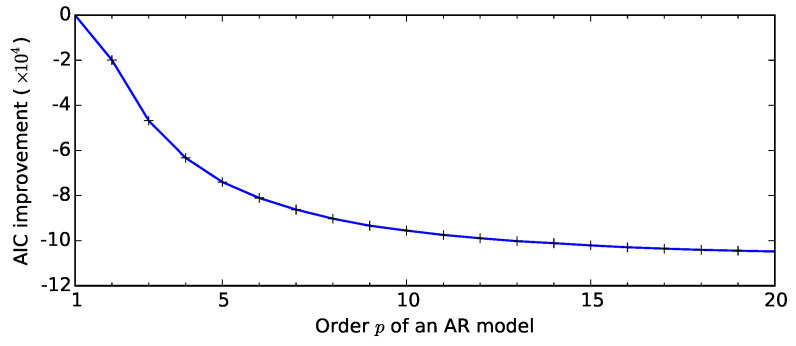
The Akaike’s Information Criterion (AIC) score for autoregressive (AR) models of increasing order using the Dunedin GPS altitude dataset recorded on 15 August 2014, scaled relative to the AIC score for p=1.

Table 2 clearly shows that the Gaussian white noise model, irrespective of the geographic location or the coordinate, is the worst-performing noise model. All of its AIC values are positive, indicating a very low likelihood that the observations are described by the model. The MA models also seem a poor fit to the data, although one may increase the order of the model, *q*, to achieve lower AIC scores. Our standard numerical routine, based on the Yule–Walker method [37], could not converge. In contrast, an AR model with increasing order fit best among the noise models examined. Although both the ARMA(*p*, *q*) and Ornstein–Uhlenbeck models could achieve comparable AIC values (with the exception of the North Stradbroke Island dataset), the quantitative comparison of AIC values always favoured the high-order AR model. We note here that slightly lower AIC values could be achieved for autoregressive models by increasing their order, but the gain is becoming negligible around p≈10 (see Figure 7).

Figure 7 also captures two typical phenomena: For low values of *p*, the AR(*p*) models do improve significantly with increasing values of *p*, but there is a ‘kink’ in the AIC curve around approximately p=10, where some improvement can be still achieved by increasing *p*, but these may not be worthwhile considering the increasing computational workload. In many GPS applications, the sampling period is often shorter than a few minutes or an hour, so the aim of developing a noise model is to focus on the short-term behaviour of the time series. For this reason, the choice of p=9 seems a good compromise between modelling performance and computational load (cf. Table 2).

In summary, the Gaussian white noise model is the worst fit to the observation. The next worst model, according to the AIC, is a low-order MA process, followed by the Ornstein–Uhlenbeck model. A higher-order AR scores better, with the higher-order AR processes scoring best. The AR process has the further advantage of robust parameter estimation.

## 6. Discussion

The use of Gaussian white noise models is very common in sensor fusion algorithms that incorporate GPS data (Hamilton 1994). We have shown here that the assumption of uncorrelated noise does not capture important features of real-world GPS data. Fitting a Gaussian white noise model results in an artificially large variance and does not account for the observed autocorrelations. Analysis of location data shows that the error between the position measurement and the ‘true’ location shows strong autocorrelation up to approximately an hour. We proposed stochastic noise models that take into account such autocorrelation. Estimating the model parameters for the Ornstein–Uhlenbeck and AR models based on likelihood maximisation is straightforward where either the analytic solution is known or the numerical algorithm is well known and can be performed efficiently. Conversely, estimating the parameters for the MA or ARMA models is more involved and computationally expensive. If the stochastic process is known to be non-stationary, then the MA model is non-invertible, and further measures are required to estimate the model parameters. Here, we mention an alternative approach for supporting the suitability of novel noise models. One can calculate the second moment of the residuals, $\langle E_{t}^{2}\rangle$, using single-path or multipath ensemble averaging for the observed time series and the one generated by the model. However, this approach is left for future analysis.

Autoregressive processes of moderate order provide a sufficient noise model for the signal returned by the commercial GPS device, and the extra computational cost compared to that of a naive Gaussian white noise model is negligible. Increasing the order of the autoregressive process results in lower AIC values, indicating that, from the purely statistical perspective, the likelihood maximisation outweighs the increase in complexity. However, for the less accurate GPS units, the higher-order AR processes are only slightly more likely than the first-order AR(1) process. For an AR(1) process, we would advise a discretised Ornstein–Uhlenbeck process, which brings several advantages. First, the sampling period of the device is explicit in its formulation; thus, variable sampling periods can easily be fit into this description. Second, the other parameters of an Ornstein–Uhlenbeck process give clear insight into the noise characteristics: θ describes how the rate measurements revert towards the mean μ, while σ indicates the amount of noise present between each measurement.

Wendel and Trommer [59] analysed the effect of time-correlated noise on the applicability of classical Kalman Filtering, and proposed new filter equations. The noise models we proposed here can thus be incorporated into a Kalman Filter or Bayesian inference algorithms and provide both an estimate for the location along with quantified uncertainty. In addition, recent work [60] has shown that there are practical algorithms for sensor fusion in multi-dimensional systems that allow arbitrary noise distributions and approach the computational efficiency of Kalman Filtering. 

## Figures and Tables

**Table 1:** Measurement sites and dates of observations.

Measurement Site	Latitude (South)	Longitude (East)	Dates
North Stradbroke Island	27^∘^25${}^{\prime }$14${}^{\prime \prime }$	153^∘^30${}^{\prime }$52${}^{\prime \prime }$	14 September 2014
Port Douglas	16^∘^29${}^{\prime }$07${}^{\prime \prime }$	145^∘^27${}^{\prime }$59${}^{\prime \prime }$	17 September 2014
Thursday Island	10^∘^34${}^{\prime }$79${}^{\prime \prime }$	142^∘^13${}^{\prime }$47${}^{\prime \prime }$	21 September 2014
Dunedin	45^∘^ 51${}^{\prime }$50${}^{\prime \prime }$	170^∘^30${}^{\prime }$48${}^{\prime \prime }$	6 August 2014
			7 August 2014
			15 August 2014
			27 September 2014
			5 October 2014
			22 October 2014

**Table 2:** AIC values (rounded) of noise models for GPS time series.

**Thursday Island**	Gauss	AR(9)	MA(1)	MA(3)	ARMA(1, 1)	OU
Latitude	368,278	−419,119	240,593		−391,014	−390,082
Longitude	490,408	−358,983	369,403		−201,441	−212,528
Altitude	616,549	−151,204	488,436		−106,982	−107,027
**Port Douglas**					ARMA(1, 1)	
Latitude	1,080,721	−1,104,629	749,275		−1,048,908	−1,031,940
Longitude	1,254,838	−415,461	927,860		−887,968	−1,016,929
Altitude	1,445,244	−844,480	1,112,076		−924,883	−659,902
**North Stradbroke**					ARMA(2,2)	
Latitude	616,991	−846,881	417,772	160,425	−54,584	4099
Longitude	552,469	−847,589	369,864	138,410	48,340	5140
Altitude	991,469	−638,245	783,784	549,365	280,719	401,359
**Dunedin**					ARMA(2,2)	OU
Latitude	2,827,919	−4,855,973				−4,762,660
Longitude	2,202,644	−5,333,179	945,600		−5,343,304	−5,292,592
Altitude	4,380,261	−4,351,240	3,112,342		−4,360,823	−4,114,385

## Appendix A. Short Description of the QQ-Plot Method for Testing Normality

Before constructing non-Gaussian noise models, it is desirable to show that the distribution of noise is significantly different from a Gaussian distribution [23]. We thus carried out three normality tests for each coordinate (latitude, longitude, and altitude): the d’Agostino–Pearson test [25], the Lilliefors test [26], and the graphical QQ-plot method [61]. In Section 2, we demonstrated the deviation of the residuals from a Gaussian distribution. Here, a short description of the QQ-plot method is provided.

The central concept of the QQ-plot method is that of the quantiles, a measurable functional of the empirical distribution, such as the sample mean and sample median. The empirical distribution function, $F_{n}$, of a sample of size *n*, is defined as $F_{n}\left(x\right)=P_{n}^{*}\left(\left(-\infty ,x\right)\right)$, or, in words, the ratio of elements of the sample that are less than *x*. The symbol * means that the probability is estimated by the observed frequency. Assuming that $F_{n}\left(x\right)$ converges in probability to a distribution function F(x) as n→∞, we can define the quantile, $\zeta_{p}$, of order *p* as ζ(p)=sup(x|F(x)≤p). Here, the usage of sup guarantees that this definition remains meaningful even if F(x) has points of discontinuity. As a function of *p*, the quantile ζ(p) is the inverse function of F(x). A widely used example of a quantile is the median, which is simply $\zeta (\frac{1}{2})$, i.e., the value $\hat{x}$ of the sample for which half of the observations are smaller than $\hat{x}$, and half of them are higher than $\hat{x}$. Similarly, we may divide up the entire range of the random variable into segments in a way that the number of samples belonging into each segment are the same. In the sample quantile $\zeta_{n}^{*}\left(p\right)=x_{\ell }$, where ℓ=[np]+1, $x_{k}$ is equal to the *k*-th smallest value of the sample.

The sample quantiles provide us a graphical approach to check whether the sample comes from a given probability distribution, G(x). Let us assume that $X_{1}$, $X_{2}$, …are independent, identically distributed random variables, with E[X]=0 and $\mathrm{Var}\left[X\right]=\sigma^{2}<\infty$. Even if the observed values do not satisfy these mild conditions, after normalisation, the two conditions can be fulfilled. Let *G* denote the distribution function of the standard normal distribution, while $F_{n}$ stands for the empirical distribution function of $S_{n}^{*}$, where

$$S_{n}^{*}=\frac{1}{\sqrt{n\sigma^{2}}}\left(X_{1}+X_{2}+\cdots \right).$$

One measure of whether the sample $\left\{x_{1},x_{2},\cdots \right\}$ comes from a standard normal distribution is the ‘closeness’ of $F_{n}$ and *G*. The closeness is visually represented in a two-dimensional plot, where the abscissa is the *t*-th quantile, ζ(t), while the ordinate is the *t*-th sample quantile, $\zeta^{*}\left(t\right)$. The closer they are, the closer the points are to the diagonal. The plot of points $\left(\zeta \left(t\right),\zeta^{*}\left(t\right)\right)$ for t∈R is called the quantile–quantile plot, or, in short, the QQ-plot. References

## References

[1] Zumberge J.F., Heflin M.B., Jefferson D.C., Watkins M.M., Webb F.H. (1997). Precise point positioning for the efficient and robust analysis of GPS data from large networks. J. Geophys. Res..

[2] Zandbergen P.A., Barbeau S.J. (2011). Positional accuracy of assisted GPS data from high-sensitivity GPS-enabled mobile phones. J. Navig..

[3] Olynik M.C. (2002). Temporal Characteristics of GPS Error Sources and Their Impact on Relative Positioning.

[4] Van Nee R.D. (1992). Multipath effects on GPS code phase measurements. Navigation.

[5] Georgiadou Y., Kleusberg A. (1988). On carrier signal multipath effects in relative GPS positioning. Manuscripta Geod..

[6] Sigrist P., Coppin P., Hermy M. (1999). Impact of forest canopy on quality and accuracy of GPS measurements. Int. J. Remote Sens..

[7] Griffiths J., Ray J.R. (2009). On the precision and accuracy of IGS orbits. J. Geod..

[8] Forte B. (2012). Analysis of the PLL phase error in presence of simulated ionospheric scintillation events. Radio Sci..

[9] Tiberius C., Jonkman N., Kenselaar F. (1999). The stochastics of GPS observables. GPS World.

[10] Xu G., Xu Y. (2016). GPS: Theory, Algorithms and Applications.

[11] Santamaría-Gómez A., Bouin M.N., Collilieux X., Wöppelmann G. (2011). Correlated errors in GPS position time series: Implications for velocity estimates. J. Geophys. Res. Solid Earth.

[12] Zhang J., Bock Y., Johnson H., Fang P., Williams S., Genrich J., Wdowinski S., Behr J. (1997). Southern California permanent GPS geodetic array: Error analysis of daily position estimates and site velocities. J. Geophys. Res. Solid Earth.

[13] Bos M., Fernandes R., Williams S., Bastos L. (2008). Fast error analysis of continuous GPS observations. J. Geod..

[14] Olivares G., Teferle F. (2013). A Bayesian Monte Carlo Markov Chain Method for Parameter Estimation of Fractional Differenced Gaussian Processes. IEEE Trans. Signal Process..

[15] Jin S., Wang J., Park P.H. (2005). An improvement of GPS height estimations: Stochastic modeling. Earth Planets Space.

[16] Borre K., Tiberius C. Time Series Analysis of GPS Observables. Proceedings of the 13th International Technical Meeting of the Satellite Division of the Institute of Navigation (ION GPS 2000).

[17] Segall P., Davis J.L. (1997). GPS applications for geodynamics and earthquake studies. Annu. Rev. Earth Planet. Sci..

[18] Amiri-Simkooei A.R. (2009). Noise in multivariate GPS position time-series. J. Geod..

[19] Williams S.D.P., Bock Y., Fang P., Jamason P., Nikolaidis R.M., Prawirodirdjo L., Miller M., Johnson D.J. (2004). Error analysis of continuous GPS position time series. J. Geophys. Res. Solid Earth.

[20] Santamaría-Gómez A., Bouin M.N., Collilieux X., Wöppelmann G. (2013). Chapter Time-Correlated GPS Noise Dependency on Data Time Period. Reference Frames for Applications in Geosciences.

[21] Kaplan E.D., Hegarty C.J. (2006). Understanding GPS Principles and Applications.

[22] Thode H. (2019). Testing for Normality.

[23] Yap B.W., Sim C.H. (2011). Comparisons of various types of normality tests. J. Stat. Comput. Simul..

[24] Pearson E.S.I. (1931). Note on Tests for Normality. Biometrika.

[25] D’Agostino R.B., Belanger A. (1990). A Suggestion for Using Powerful and Informative Tests of Normality. Am. Stat..

[26] Lilliefors H.W. (1967). On the Kolmogorov-Smirnov Test for Normality with Mean and Variance Unknown. J. Am. Stat. Assoc..

[27] Abdel-Hafez M.F. (2010). The autocovariance least-squares technique for GPS measurement noise estimation. IEEE Trans. Veh. Technol..

[28] Fallahi K., Cheng C.T., Fattouche M. (2012). Robust positioning systems in the presence of outliers under weak GPS signal conditions. IEEE Syst. J..

[29] Durbin J. (1960). The Fitting of Time-Series Models. Rev. Int. Stat. Inst..

[30] Durbin J. (1960). Efficient Fitting of Linear Models for Continuous Stationary Time Series from Discrete Data.

[31] Durbin J. (1961). Efficient fitting of linear models for continuous stationary time series from discrete data. Bull. Int. Stat. Inst..

[32] Cryer J.D., Chan K.S. (2008). Time Series Analysis: With Applications in R.

[33] Box G.E.P., Jenkins G.M., Reinsel G.C., Ljung G.M. (2016). Time Series Analysis: Forecasting and Control.

[34] Walker A.M. (1962). Large-Sample Estimation of Parameters for Autoregressive Processes with Moving-Average Residuals. Biometrika.

[35] Box G.E.P., Pierce D.A. (1970). Distribution of Residual Autocorrelations in Autoregressive-Integrated Moving Average Time Series Models. J. Am. Stat. Assoc..

[36] Ljung G.M., Box G.E.P. (1978). On a measure of lack of fit in time series models. Biometrika.

[37] Box G.E.P., Jenkins G.M., Reinsel G.C. (1994). Time Series Analysis, Forecasting and Control.

[38] Bartlett M.S. (1946). On the theoretical specification of sampling properties of an autocorrelated process. J. R. Stat. Soc. B.

[39] Anderson O. (1976). Time Series Analysis and Forecasting: The Box-Jenkins Approach.

[40] Fishman G. (1978). Principles of Discrete Event Simulation.

[41] Porat B. (1994). Digital Processing of Random Signals: Theory and Methods.

[42] Akaike H. (1973). Maximum likelihood identification of Gaussian autoregressive moving average models. Biometrika.

[43] Brockwell P.J., Davis R.A. (1991). Time Series: Theory and Methods.

[44] Astrom K., Soderstrom T. (1974). Uniqueness of the maximum likelihood estimates of the parameters of an ARMA model. IEEE Trans. Autom. Control.

[45] Mann H.B., Wald A. (1943). On the Statistical Treatment of Linear Stochastic Difference Equations. Econometrica.

[46] Melard G. (1984). Algorithm AS197: A Fast Algorithm for the Exact Likelihood of Autoregressive-Moving Average Models. J. R. Stat. Society. Ser. C Appl. Stat..

[47] Evans L.C. (2014). An Introduction to Stochastic Differential Equations.

[48] Øksendal B. (2010). Stochastic Differential Equations: An Introduction with Applications.

[49] Uhlenbeck G.E., Ornstein L.S. (1930). On the Theory of the Brownian Motion. Phys. Rev..

[50] Doob J.L. (1942). The Brownian Movement and Stochastic Equations. Ann. Math..

[51] Pedersen A.R. (1995). A New Approach to Maximum Likelihood Estimation for Stochastic Differential Equations Based on Discrete Observations. Scand. J. Stat..

[52] Tang C.Y., Chen S.X. (2009). Parameter estimation and bias correction for diffusion processes. J. Econom..

[53] Dacunha-Castelle D., Florens-Zmirou D. (1986). Estimation of the coefficients of a diffusion from discrete observations. Stochastics.

[54] Kloeden P.E., Platen E. (1992). Numerical Solution of Stochastic Differential Equations.

[55] Claeskens G., Hjort N.L. (2008). Model Selection and Model Averaging.

[56] Akaike H. (1974). A new look at the statistical model identification. IEEE Trans. Autom. Control.

[57] Schwarz G. (1978). Estimating the Dimension of a Model. Ann. Stat..

[58] Hastie T., Tibshirani R., Friedman J. (2003). The Elements of Statistical Learning: Data Mining, Inference, and Prediction.

[59] Wendel J., Trommer G.F. An Efficient Method for Considering Time Correlated Noise in GPS/INS Integration. Proceedings of the 2004 National Technical Meeting of The Institute of Navigation.

[60] Fox C., Norton R.A., Morrison M.E., Molteno T.C. (2019). Sequential Bayesian Inference for Dynamical Systems Using the Finite Volume Method. 2017 MATRIX Annals.

[61] Thode H. (2002). Testing for Normality.

